# Involvement of IL-4, IL-13 and Their Receptors in Pancreatic Cancer

**DOI:** 10.3390/ijms22062998

**Published:** 2021-03-15

**Authors:** Jingwei Shi, Xujun Song, Benno Traub, Michael Luxenhofer, Marko Kornmann

**Affiliations:** Department of General and Visceral Surgery, Ulm University Hospital, Albert-Einstein-Allee 23, 89081 Ulm, Germany; shijingwei555@126.com (J.S.); 101012268@seu.edu.cn (X.S.); benno.traub@uniklinik-ulm.de (B.T.); michael.luxenhofer@uni-ulm.de (M.L.)

**Keywords:** interleukin-4, interleukin-13, interleukin-4 receptor, interleukin-13, cytokine, pancreatic cancer

## Abstract

Interleukin (IL)-4 and IL-13 are known as pleiotropic Th2 cytokines with a wide range of biological properties and functions especially in immune responses. In addition, increasing activities have also been determined in oncogenesis and tumor progression of several malignancies. It is now generally accepted that IL-4 and IL-13 can exert effects on epithelial tumor cells through corresponding receptors. Type II IL-4 receptor (IL-4Rα/IL-13Rα1), predominantly expressed in non-hematopoietic cells, is identified to be the main target for both IL-4 and IL-13 in tumors. Moreover, IL-13 can also signal by binding to the IL-13Rα2 receptor. Structural similarity due to the use of the same receptor complex generated in response to IL-4/IL-13 results in overlapping but also distinct signaling pathways and functions. The aim of this review was to summarize knowledge about IL-4 and IL-13 and their receptors in pancreatic cancer in order understand the implication of IL-4 and IL-13 and their receptors for pancreatic tumorigenesis and progression and for developing possible new diagnostic and therapeutic targets.

## 1. Introduction

Despite advances in treatment, cancer is globally the second-leading cause of death [1,2]. The 5-year relative survival rate is lowest for cancers of the pancreas due to aggressive local growth combined with the rapid development of distant metastases and very limited improvement of surgical and medical treatments over recent decades [3]. Therefore, innovative diagnostic and therapeutic options are desperately needed for the management of this dismal disease. To reach this target it is vital to understand pancreatic cancer development and progression, in which the tumor microenvironment (TME) has received significant attention [4,5,6,7]. Many studies have identified heterogeneous components of the TME in epithelial cancers, containing fibroblasts of several phenotypes, extracellular matrix, immune and inflammatory cells, blood and lymph vessels, and nerves [8,9,10], all capable of influencing malignant behavior [11,12]. Among the immune cells recruited to a tumor site, tumor-associated macrophages (TAMs) are particularly plentiful and are present at all stages of tumor progression [13,14]. Polarized in the environment of chronic inflammation, M2-like phenotype TAMs [15,16] have been shown to facilitate angiogenic responses, promote tumor proliferation, and ultimately bring out tumor metastasis, instead of diminishing inflammation and helping to eradicate tumor cells [17,18,19]. In addition, the presence of specific receptors and the production of cytokines by the tumor cells as well as the surrounding microenvironment [20,21,22] have been directly linked to aggressive tumor growth, invasion, metastasis, and suppression of tumor-directed immune surveillance mechanisms [23,24,25].

Considering the cytokines produced in the TME, the role of the interleukin-4 (IL-4)/interleukin-13 (IL-13) cytokine-receptor system [26,27,28] has shown significant influence on cancer cell survival, progression, and metastasis [29,30,31]. The aim of this review was to summarize the present knowledge about the IL-4/IL-13 cytokine-receptor system focusing on pancreatic cancer to help to develop attractive targets for novel diagnostic and therapeutic approaches for pancreatic cancer.

## 2. Methods

**Figure 1:**
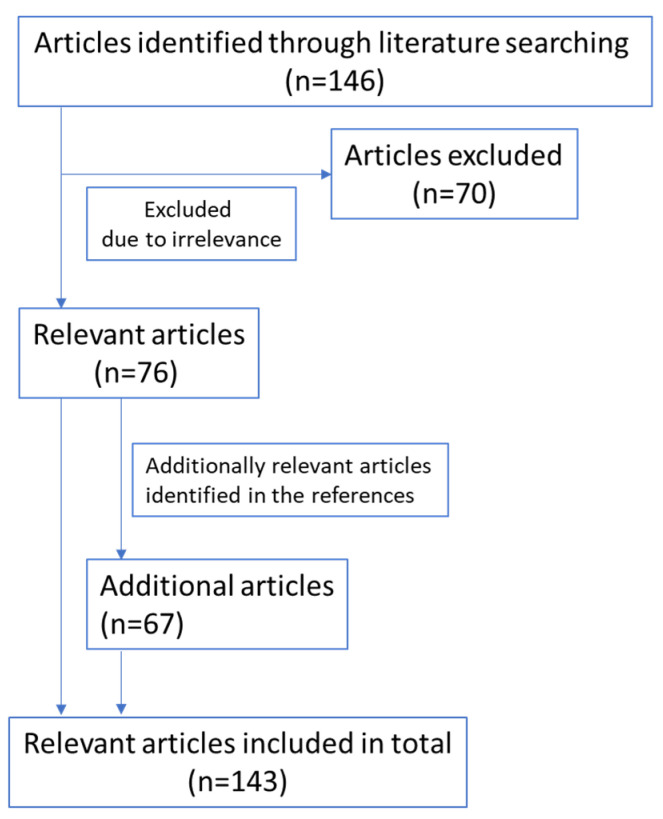
Relevant references included.

A literature search was performed in PubMed in May 2020 by using the terms “IL-4”, “interleukin-4”, “IL-13”, “interleukin-13”, “IL-4R”, “interleukin-4 receptor”, “IL-13R”, or “interleukin-13 receptor” in combination with “pancreatic cancer”. In total, 146 articles were identified through this database search. After screening the titles and abstracts, 76 articles related to the topic were included, while 70 articles were excluded because of irrelevance. An additional 67 articles were identified through references cited in the retrieved articles. Only abstracts, manuscripts, and reviews in the English language were included in this review. The flow-chart of relevant references included is as follows (Figure 1):

## 3. IL-4 and IL-13 Cytokines and Their Receptors

IL-4 and IL-13, encoded by adjacent genes and sharing similar transcriptional regulatory elements [32], are widely recognized as pleiotropic Th2 cytokines [33,34] with 25–30% sequence homology and many similar characteristics [35,36]. Studies demonstrated that IL-4 and IL-13, secreted by epithelial cells, lymphocytes, eosinophils, basophils, and mast cells [37,38,39,40,41], have a wide range of overlapping but also distinct biological functions, particularly in inflammatory and allergic diseases [41,42]. Nevertheless, increasing activities of IL-4 and IL-13 in cancers such as lymphoma [43,44], breast [45,46], lung [19], colorectal [47,48], and oral squamous cell [49], as well as pancreatic [50] have been found closely associated with tumorigenesis and metastasis. It has been determined that the overexpression of IL-4 and/or IL-13 in the microenvironment of carcinomas, produced by stroma or tumor cells can promote tumor progression in autocrine and paracrine ways [51,52,53,54] through multiple mechanisms such as stimulating the polarization of macrophages to the alternatively activated M2 phenotype [16,55], initiating oncogenesis [56], enhancing survival via mediating resistance to apoptosis and strengthening metabolism [30,53,57,58], facilitating proliferation, migration, and invasion via participating in intricated pathways [54,59,60,61], and increasing the metastatic tumor burden [18,62].

**Figure 2:**
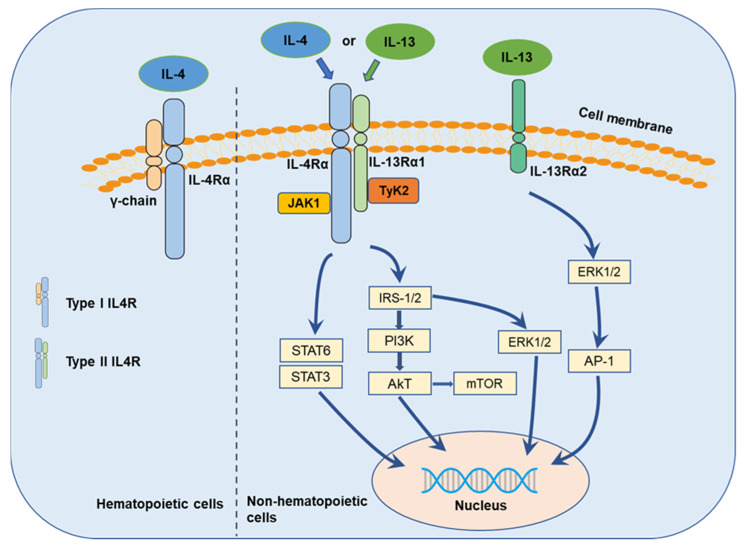
IL-4/IL-13 axis and their signaling transduction.

The multiple functions of IL-4 are initiated through the binding of IL-4 to its respective transfer membrane receptor chain (Figure 2), IL-4-receptor (IL-4R), in both the type I receptor complex, comprising of IL-4-receptor alpha (IL-4Rα) and the common gamma chain (γc) (IL-4/IL-4Rα/γc) [41], predominantly expressed on hematopoietic cells, and the type II receptor complex, comprising of IL-4Rα and IL-13-receptor alpha (IL-13Rα) 1 (IL-4/IL-4Rα/IL-13Rα1) [63,64], predominantly expressed on non-hematopoietic cells [65]. IL-13 is commonly identified to bind to two different IL-13-receptors, IL-13Rα1 and IL-13Rα2 [66]. IL-13Rα1, recruited to IL-4Rα, binds IL-13 with high affinity and forms a functional receptor for IL-13 (IL-13/IL-13Rα1/IL-4Rα) [63,66]. Thus, the type II IL-4R is generated by the binding of IL-13 to IL-13Rα1 and subsequent heterodimerization with IL-4Rα. IL13Rα2 was hypothesized to be a decoy receptor for IL13Rα1 and the type II IL-4R complex [67,68], but updated evidence suggests that IL-13 may signal through IL-13Rα2 to promote pancreatic cancer cell proliferation and invasion [68,69,70].

In response to IL-4/IL-13, the diverse receptor heterodimers induce the phosphorylation of janus tyrosine kinases (JAKs, JAK1, and JAK2) or tyrosine kinase 2 (Tyk2) [71,72], which activates further downstream signaling (Figure 2). The proline-rich box regions in the intracellular domain of the receptors thereby mediate association with the JAKs instead of exhibiting an intrinsic kinase activity [73]. Signal transducer and activator of transcription (STAT)6 [74], insulin receptor substrate (IRS)/phosphoinositide 3-kinase (PI3K)/protein kinase B (AKT) [75], IRS/extracellular signal-regulated kinase (ERK) [41], and the mechanistic target of rapamycin (mTOR) [62] are supposed to be the main downstream signaling pathways [52,76]. In addition, IL-13 appears to play its own distinct role in cancer cells through binding to IL-13Rα2 with high affinity [77] and mediating invasion and metastasis via IL-13Rα2, ERK/activator protein 1 (AP-1) signaling, and matrix metalloproteinases (MMPs) pathways [78]. Besides, Song summarized the structure of IL-4R, IL-13R, and the downstream complex web where IL-4/IL-13 initiated signal transduction through three types of IL-4 receptors and three types of IL-13 receptors [79].

## 4. IL-4 in Pancreatic Cancer

It was reported that the IL-4 protein level was significantly higher in the plasma of pancreatic ductal adenocarcinoma (PDAC) patients compared with control participants [80,81]. Piro et al. demonstrated that circulating IL-4 was an independent prognostic factor for disease-free survival in PDAC patients after surgical resection, where a level of IL-4 higher than a defined cutoff was significantly associated with worse prognosis [82]. These data indicate the possible association between the excessive presence of IL-4 and PDAC development and progression. Expression of endogenous IL-4 has also been determined in total cell lysates from pancreatic cell lines COLO-357, MIA PaCa-2, PANC-1, ASPC-1, Capan-1, and T3M4 on the protein level by ELISA, and on the mRNA level by real-time PCR analysis [54], which points to possible autocrine and paracrine actions in pancreatic cancer.

Studies demonstrated that IL-4 exerts stimulating effects on pancreatic cancer cell proliferation and survival. For example, previous work showed that exogenous human IL-4 (5 nM) enhanced the growth of COLO-357 [50], and further studies confirmed that IL-4 exerted dose-dependent increases in the growth of four other cultured pancreatic cancer cell lines [54]. Considering the close relationship of IL-4 with TAMs, there is little surprise that IL-4 was shown to induce the ability of TAM-derived cathepsin protease, leading to cancer progression, invasion, and angiogenesis [83]. In an indirect coculture system, M2-polarized TAMs induced by IL-4-treatment enhanced the malignant phenotypes of pancreatic cancer cells, promoting epithelial–mesenchymal transition (EMT), and eventually leading to increased cell proliferation and migration [84]. These findings support the dual role of IL-4 exerting paracrine functions in pancreatic cancer tissue in addition to autocrine actions. Furthermore, the interactions of IL-4 with other cytokines appear to generate synergistic effects for tumorigenesis. Wu et al. reported that IL-4 alone or combined with IL-17A strongly induced the expression of dual oxidase 2 and its cognate maturation factor, leading to long-lasting H_2_O_2_ production and DNA damage in pancreatic cancer cells, while increased expression of dual oxidase 2 and IL-4R in clinical tumor tissues was conversely associated with overall patient survival [85]. IL-6 was found to stimulate the cancer-promoting macrophage phenotype change through regulating the level of receptors for IL-4 [86]. In addition, IL-4-blockade had a significantly inhibitory impact on pancreatic cancer progression, where IL-4 neutralizing antibody was proven to inhibit the basal growth of COLO-357, PANC-1, and MIA PaCa-2 cells [54]. Even more, suppression of IL-4 mRNA in the liver of cachexia patients with pancreatic cancer [87] and improved performance of carcinoma-bearing mice treated by IL-4 [88] may provide beneficial approaches for pancreatic cancer patients suffering from tumor-induced cachexia in the future.

It is important to understand the signaling pathways of IL-4 in pancreatic cancer as possible therapeutic targets. Pancreatic cancer cells were determined to express the transcription factors STAT1, STAT3, and STAT6 at various levels, while Stat3 phosphorylation was enhanced in response to IL-4 stimulation [54], and STAT6 nuclear translocation was increased after exposure to IL4 [85]. IRS-2 was found to overexpress in human pancreatic cancer and might stimulate tumor growth through enhancing mitogenic signaling via the PI3-kinase pathway [89]. Prokopchuk et al. showed that IL-4 induced strong tyrosine phosphorylation of IRS-1 and IRS-2 and enhanced mitogen-activated protein kinase (MAPK) and Akt activity [54]. In addition, Traub et al. determined that the strong phosphorylation of pro-oncogenic pathways containing c-Jun, ERK-1/2, and STAT3 in Capan1 cells were induced by exogenous IL-4 stimulation [90], while the specific molecular inhibitor of STAT3 phosphorylation LLL12 [91], showed inhibition of pancreatic cancer cell survival.

## 5. IL-13 in Pancreatic Cancer

A significant elevation of IL-13 protein detected in the plasma of pancreatic cancer patients was reported [92]. In addition, increased IL-13 levels were correlated with elevated levels of myeloid-derived suppressor cells that were associated with increased risk of death from pancreatic cancer. IL-13 protein has also been detected in both total cell lysates and conditioned medium of COLO-357, MIA PaCa-2, PANC-1 ASPC-1, Capan-1, and T3M4 cells by ELISA, while the presence of IL-13-mRNA transcript was examined by Northern blotting [93], indicating that pancreatic cancer cells can produce and also secrete IL-13 to exert autocrine and paracrine effects. In addition, high IL-13 immunoreactivity was determined in the ductal cancer cells in 43% (30 of 70) of pancreatic cancer tissues [93]. IL-13 immunoreactivity was not present in normal ductal, acinar, or islet cells [93].

It has also been shown that IL-13 enhanced the growth of ASPC-1, Capan-1, and COLO-357 cells in a dose-dependent manner along with the percentage of cells increased in S-phase and reduced in G0/G1 [50,93]. There was no correlation between the expression level of IL-13-receptor and cell proliferation induced by exogenous IL-13 [50,93], which indicates the intricate interactions between IL-13 with its receptors may exert complicated effects on proliferation depending on cancer cell types. Moreover, it was reported that synthetic thyalpha1 promoted PANC-1 cell proliferation with increased secretion of IL-13 [94], which further confirmed the close association between IL-13 with pancreatic cancer cell growth. Besides the direct mitogenic effects of IL-13 on pancreatic cancer cells, tumor-cell-derived IL-13 along with IL-13 produced by other cells in the TME-like mast cells [95] stimulated the proliferation of pancreatic stellate cells (PSCs). PSCs are known to participate in successfully reducing the effect of cancer-cell directed therapeutic drugs and regulating the interactions of immunosuppressive cells with stromal cells, overall promoting the growth of pancreatic cancer. In this context, IL-13 was also identified to induce tissue fibrosis in the liver [92] and lung [96,97] with the involvement of AP-1, transforming growth factor beta 1 (TGF-β1) and IL-13Rα2. Furthermore, IL-13 secreted by activated PSCs was found to initiate the polarization of TAMs in the TME [98], to promote pancreatic fibrosis, and to mediate pancreatic tumorigenesis [99]. Thus, there is the possibility that IL-13, in addition to imposing direct stimulating effects on pancreatic cancer cell progression, may also contribute to the inhibition of anti-tumor immune mechanisms, thereby facilitating tumor spread. Similar to the blockade of IL-4, incubation of ASPC-1 and Capan-1 cells with increasing concentrations of IL-13 neutralizing antibody showed an inhibitory effect on cell growth in a dose-dependent manner, while the mitogenic activity of IL-13 was significantly suppressed due to preincubation with the neutralizing antibody in Capan-1 cells [93]. In addition, the treatment of mast-cell-conditioned medium with neutralizing anti-IL-13 antibody showed a suppressive impact on the proliferation of pancreatic stellate cells [95].

It has been shown that IL-13 promoted pancreatic cancer cell proliferation in association with the increased phosphorylation of p44/42 MAPK (ERK1/2) in ASPC-1, Capan-1, and COLO-357 cells, and that both the tyrosine phosphorylation of IRS-1 and IRS-2 and PI3-kinase activity was enhanced by IL-13 in pancreatic cell lines [93]. Li et al. demonstrated that thymosinalpha1 stimulated pancreatic cancer cell proliferation with the increase in IL-13 accompanying the activation of ERK1/2 and c-Jun N-terminal kinase (JNK) [94]. Furthermore, it was determined that IL-13 stimulation induced the activation of AP-1 transcription factors like c-Fos, c-Jun, and Fra-2 [100] involved in inducing TGF-β1 promoter activity, and ERK1/2 [90], which acted as upstream cytokine of the AP-1/MMP pathways through IL-13Rα2. Exogenous IL-13 was shown to induce the expression of MMPs including MMP-9, MMP-12, and MMP-14, which were related to pancreatic cancer invasion in IL-13Rα2-positive pancreatic cancer cells independent of STAT6 phosphorylation [101].

**Figure 3:**
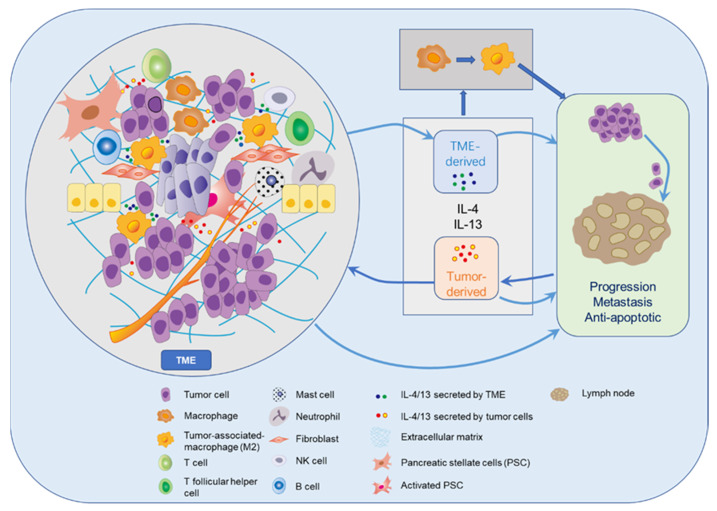
Tumor microenvironment (TME) and role of IL-4/IL-13 in tumor development.

The possible roles of IL-13 and IL-4 in pancreatic cancer within the TME and metastatic spread are summarized in Figure 3. IL-4 and IL-13 can display a stimulating influence on tumor progression and metastasis through interactions with various cells in the TME including TAMs [19,21,47]. Irrespective of their original functions IL-4 and IL-13 cytokines are capable of promoting tumor cell growth via autocrine and paracrine mechanisms, while inhibiting attacking immune cells at the same time. Interestingly, 15 of 16 (94%) specimens resected from PDAC patients exhibiting high-level co-expression of IL-13 and IL-4R had lymph node metastases [93], which reveals that IL-13 in conjunction with IL-4R in the pancreatic cancer cells seems to facilitate lymph node metastasis. During the process of metastatic spread circulating tumor cells may find optimal environmental conditions in surroundings rich with these cytokines.

## 6. IL-4R in Pancreatic Cancer

The overexpression of IL-4R in cultured pancreatic cancer cell lines and in tumor specimens resected from pancreatic cancer patients has been determined by different research groups. Therefore, IL-4R might be targeted for pancreatic cancer therapy. Our group demonstrated the expression of IL-4Rα in pancreatic cancer cell lines ASPC-1, Capan-1, MIA PaCa-2, COLO-357, PANC-1, and T3M4 by Northern blot and Western blot (WB) analysis [50]. Furthermore, it was demonstrated that not only AsPC-1, Capan-1, MIAPaCa-2, COLO-357, PANC-1, and T3M4 but also BxPC-3, expressed IL-4Rα at various levels [90]. RNA expression of IL-4Rα was also detected in AsPC-1 and BxPC-3 cells by quantitative RT-PCR [85]. Shimamura et al. reported that six of eight examined pancreatic cancer cell lines expressed various levels of IL-4Rα mRNA, whereas human pancreatic duct epithelial cells showed no expression [102]. Kawakami et al. determined that IL-4R was overexpressed not only in the membrane of cultured pancreatic cancer cells, but also in tumor samples derived from patients diagnosed with pancreatic cancer and was barely present in normal pancreatic tissues [103]. The existence of high IL-4R immunoreactivity was detected in the ductal cancer cells in 40% (28 of 70) of primary PDAC samples [93]. Immunohistochemical analysis for the expression of IL-4Rα in PDAC specimens showed 60% (42 of 70) cases expressing moderate to high levels of IL-4Rα, whereas only weak staining for IL-4Rα was observed in 2 of 15 (13%) normal pancreas tissues [102]. Immuno-fluorescence staining showed an increase not only in the expression of IL-4Rα in pancreatic cancer cells, but also in the M2 macrophages expressing IL-4Rα in the samples from pancreatic cancer patients compared with normal tissues [86]. Thus, it is not surprising that IL-4Rα is involved in the etiology of pancreatic cancer as a risk factor, where a variant of IL-4 (G3017T) might influence the risk of pancreatic cancer development according to the presence of allergies [104].

Cell surface receptors provide targets for tumor therapies like cytotoxins and immunotoxins, which have the advantage of improved specificity and direct toxicity to tumor cells overexpressing the receptors with limited toxicity to normal tissues. That IL-4Rα is overexpressed in pancreatic cancer and downregulation of IL-4Rα by shRNA plasmids resulted in reduced cell growth and migration abilities, combining the impaired IL-4 signaling in pancreatic cancer cells and inhibition on subcutaneous xenograft tumors [90], suggests that IL-4Rα may serve as an attractive target for novel approaches to treating pancreatic cancer. Recombinant IL4-*Pseudomonas* exotoxins (IL-4-PE), like IL-4-PE38QQR [50] and IL4(38-37)-PE38KDEL [103], were shown to suppress the progression of pancreatic cancer in vivo and in vitro. Another IL-4 cytotoxin, composed of IL-4 and truncated *Pseudomonas* exotoxin, exhibited specifical and efficient cytotoxicity to pancreatic cancer cells and when combined with gemcitabine showed synergistic anti-tumor activity in vitro and in metastatic and orthotopic mouse models [102]. Molecules targeting the combination of receptors for cytokines may show efficient toxicity in cancer. Mohammed et al. described that the transgenic expression of a molecule comprised of IL-4-receptor exodomain linked to IL-7-receptor endodomain in a chimeric antigen receptor–prostate stem cell antigen T cells inverted the inhibitory effects of IL-4 on T cell proliferation, and then reversed immunosuppressive TME, leading to the depression of tumor activity in vitro and in vivo [105]. In addition, a hybrid peptide (IL-4Rα-lytic) containing a target moiety to bind to IL-4Rα and a cellular toxic lytic peptide that selectively kills cancer cells showed anticancer potential in pancreatic cancer cell lines expressing IL-4Rα and in a xenograft mice model of BXPC-3 cells [106].

## 7. IL-13R in Pancreatic Cancer

Several studies have shown the excessive existence of the IL-13Rα1 and IL-13Rα2 chains in pancreatic cancer [50,90,100,107,108]. The expression of IL-13Rα1 in pancreatic cancer cells including ASPC-1, Capan-1, MIA PaCa-2, COLO-357, PANC-1, T3M4, and BxPC-3 was determined on both protein and mRNA levels [50,90,107]. It has been demonstrated that high levels of IL-13Rα2 mRNA were expressed in SW1990, MIA-PaCa-2, KLM, HS766T, and BxPC3 pancreatic cancer cell lines [100,108], while extremely low expression of IL-13Rα2 was examined in normal pancreatic cells including fibroblasts and ductal epithelial cell lines. Moderate-to-high density of IL-13Rα2 was found in 52 of 73 (71%) PDAC samples, while only weak staining of IL-13Rα2 was shown in normal acinar and ductal cells [100]. In addition, it has also been detected that higher levels of IL-13Rα2 were expressed in lymph node metastasis [101] and areas of perineural invasion [109], which indicates that IL-13Rα2 may be associated with invasion and metastasis in pancreatic cancer.

The fact that silencing of IL-13Rα2 inhibited invasion of HS766T cells in a Matrigel invasion assay [101] points out that IL-13Rα2 may be a therapeutic target for pancreatic cancer treatment. Anti-tumor abilities of IL-13 cytotoxins have been shown in vivo, particularly in IL-13Rα2-positive pancreatic cancer cell lines, and also in animal models of human pancreatic cancer [100,108,110]. IL13-PE displayed significant inhibition on tumor growth, leading to longer survival time, in both orthotopic and xenograft mouse models of pancreatic cancer [100]. Furthermore, gene transfer of IL-13Rα2 into tumors dramatically sensitized tumors to IL-13 cytotoxin therapy [111,112,113,114], which was also observed in pancreatic cancer [115]. Similar to the synergistic anti-tumor activity of IL-4 cytotoxin and gemcitabine, the combination of IL-13 cytotoxin with gemcitabine exhibited a remarkable and specific anti-tumor impact in pancreatic cancer cells and advanced pancreatic cancer animal models [116]. In addition, it was reported that bispecific ligand-directed toxins DTEGF13 (catalytic fragment of diphtheria toxin linked to human EGF and IL-13) had high efficacy and decreased toxicity in PANC-1 and MIAPaCa-2 cells and in a mouse model of human pancreatic cancer [117,118]. IL-13E13K, in which a glutamic acid (E) residue at position 13 was substituted by a lysine (K) residue, was shown to competitively inhibit cell proliferation and signal transduction induced by IL-4/IL-13 through preventing the formation of type II IL-4R and the phosphorylation of STAT6 [119,120].

## 8. Future Directions of Research

The overexpressed IL-4/IL-13 cytokine-receptor system in cancers including pancreatic cancer may provide an attractive target for novel diagnostic and prognostic tools. For example, IL-4 was considered to be closely related to the poor outcome of breast cancer according to the correlation between hormone receptor negativity and an increase in IL-4 in patients who died from breast cancer [121]. Increased IL-13Rα2 expression might be an independent prognostic factor for decreased overall survival in gastric cancer patients after surgical resection [122]. In addition, the polymorphisms of IL-4R involved in the etiology of pancreatic cancer have been examined [104]. Consequently, increased levels of protein and mRNA of the IL-4/IL-13-receptor axis may be useful biomarkers for disease activity and prognosis in patients with pancreatic cancer [123,124,125].

Increasing evidence supports the critical roles for IL-4 and IL-13 in the progression of pancreatic cancer. The mechanisms of how the IL-4/IL-13 cytokine-receptor system can influence the pathogenesis of other cancers may also provide new insights for further investigating their roles in pancreatic cancer. Todaro and colleagues discovered that stem-like colon tumor cells produced and utilized IL-4 to protect themselves from apoptosis [57], which signposts that the correlation of IL-4 with stem-like tumor cells in pancreatic cancer has to be taken into account. They also found that tumor-derived IL-4 increased the expression levels of antiapoptotic proteins and prevented cell death upon TRAIL exposure and chemotherapy in primary epithelial cancer cells from colon, breast, and lung carcinomas, while IL-4 blockade sensitized them [53]. Shirota et al. determined that IL-4 from T follicular helper cells downregulated antitumor immunity by inducing myeloid cells to differentiate into M2 macrophages [126], corroborating the cooperation of IL-4 and TAMs in modulating tumor progression in the TME. In addition to the signaling transduction mentioned above multiple tumor-promoting functions mediated by IL-4 are supposed to be triggered by the activation of transcription factors like T-box 21 in lung carcinogenesis [127]. IL-4-induced gene 1 selectively expressed by regulatory B cells was determined to promote B-cell-mediated immunosuppression in melanoma progression [128]. Moreover, it has been demonstrated that downregulation of IL-4/IL-13 receptors showed suppression of tumor activity in other cancers. Guo et al. showed that downregulation of IL-4R led to enhanced apoptosis, diminished proliferation, and reduced invasion of hepatocellular carcinoma cells, and abolished IL-4-induced activation of JAK/STAT6 and JNK/ERK1/2 signaling pathways [129]. Venmar and colleagues reported that IL-4Rα-downregulation decreased metastatic capacity in breast cancer [62]. Hsi and coworkers demonstrated that IL-13Rα2 knockdown with siRNA dramatically induced 15-lipoxygenase-1 expression, promoted apoptosis, and reduced tumor growth in glioblastoma [130]. In addition, Jain et al. found that direct IL-13Rα2-downregulation decreased cellular proliferation and invasion of adrenocortical carcinoma cells [131].

Considering the overexpression of IL-4/IL-13 and their respective receptors in cancers, their stimulative roles for tumor progression [53,60,132] coupled with the property that cytokines bind to respective receptors with high efficacy and specificity, it is reasonable to design novel therapeutic approaches targeting the IL-4/13 axis. IL-4/IL-13 neutralizing antibodies and IL-4/13 cytotoxins utilizing their tight connection with ligands or receptors to improve the efficiency of molecular drugs with degraded toxicity to normal tissues [133,134,135] are thought to be appealing options. Actually, several clinical studies have been performed to assess the safety and efficiency of these molecular drugs [136,137].

Ito et al. demonstrated that IL-4 neutralizing antibodies enhanced anti-tumor immunity, delayed tumor progression, and synergistically augmented cancer immunotherapies [26]. DeNardo and coworkers showed that mice treated with murine IL-4 neutralizing antibodies exhibited decreased numbers of metastatic foci in the lungs and overall attenuation of total pulmonary metastasis of mammary adenocarcinomas [18]. Surana et al. determined that IL-4 neutralization enhanced the efficacy of monoclonal antibody trastuzumab by influencing the phenotype of myeloid cells in the TME, which suggests neutralization of IL-4 in the TME takes part in suppressing generation of the productive antitumor immune response [51]. Balyasnikova et al. determined that a novel anti-IL-13Rα2 antibody improved the survival of mice intracranially implanted with a human U251 glioma xenograft [138]. Takenouchi et al. showed that combination of anti-IL-13Rα2 with DNA methyltransferase inhibitor, 5-aza-2′-deoxycytidine, which augmented IL-13Rα2 expression with epigenetic modulation in malignant mesotheliomas, significantly prolonged the survival of mice with mesothelioma xenografts [139].

In addition to neutralizing antibodies, it has been determined that IL13Rα2 D1 peptide inhibited the viability and mobility of metastatic colorectal and glioblastoma cancer cells treated with IL-13, while the enantiomer D-D1 peptide significantly increased survival in vitro [140]. In addition, IL-4-binding fusion protein APG598 and IL-4R antagonist APG201 (R121D/Y124D) improved the chemosensitivity of Hodgkin lymphoma cells [141], which indicates that the combination of classical chemotherapy with IL-4/IL-13 antagonists may improve the efficacy of the both in cancer treatments. To inhibit the aggressive tumor behavior enhanced by radiation-induced IL-4, Kim and colleagues downregulated the expression of IL-4 by miR-320/429 [136], which indicates that combining radiotherapy with IL-4-inhibiting treatment may provide an efficient strategy for decreasing post-radiation recurrence and metastasis.

Instead of blocking the binding of cytokines to receptors, targeting these receptors with cytotoxins or/and the molecules mentioned above is an attractive method for the development of promising cancer therapies, the safety and efficiency of which should be carefully monitored. In a phase II study, it was demonstrated that recombinant human IL-4 was tolerated by patients as subcutaneous administration [136]. The safety and tolerability of IL-4 cytotoxin in patients with various advanced solid tumors were determined in phase I clinical trials [137]. Clinical trials determined that direct infusion of IL-4(38-37)-PE38KDEL into recurrent malignant high-grade gliomas showed activity and safety, without systemic toxicity [142]. Results from a phase I trial in patients with metastatic adrenocortical carcinoma showed that systemic intravenous infusion of IL-13-PE was safe at 1 μg/kg, while high levels of neutralizing antibodies against PE were found in serum samples of all patients tested [143].

## 9. Conclusions

IL-4 and IL-13, produced by multiple components in the TME, mediate a wide range of functions in a variety of cancers through appropriate receptors. The IL-4/IL13-receptor axis is believed to be overexpressed and play an important role in pancreatic cancer. This roles include participating in inducing neoplasm occurrence, promoting cancer cell proliferation, and producing apoptotic resistance. In view of studies determining that both cytokines exhibit effects on tumor progression dependent on cell type and amounts of receptors expressed on the cell surface, individualized therapies should be designed for patients, which may directly target cytokines or the receptor–ligand interactions. Furthermore, the combination of inhibiting the IL-4/IL-13-receptor axis with chemotherapeutics, radiotherapy, and/or other small inhibiting molecules may provide attractive possibilities with high efficiency and specificity for pancreatic cancer treatment. In fact, clinical trials have demonstrated the safety and efficacy of several cytotoxins targeting IL-4/IL-13 receptors, although further research is needed to decrease their toxicity to normal tissues.

## Data Availability

Not applicable.

## Abbreviations

AKT	Protein kinase B
AP-1	Activator protein 1
ELISA	The enzyme-linked immunosorbent assay
EMT	Epithelial–mesenchymal transition
ERK	Extracellular signal-regulated kinase
IL	Interleukin
IL-4Rα	IL-4-receptor alpha
IL-13Rα	IL-13-receptor alpha
JAK	Janus tyrosine kinase
JNK	c-Jun N-terminal kinase
MAPK	Mitogen-activated protein kinase
MMPs	Matrix metalloproteinases
mTOR	The mechanistic target of rapamycin
PCR	Polymerase chain reaction
PDAC	Pancreatic ductal adenocarcinoma
PE	Pseudomonas exotoxins
PSCs	Pancreatic stellate cells
Γc	Common γ chain
RNA	Ribonucleic acid
STAT	Signal transducer and activator of transcription
TAMs	Tumor-associated macrophages
TME	Tumor microenvironment
Tyk2	Tyrosine kinase 2
